# Mediating effects of physical activity enjoyment on physical activity levels in adults with cystic fibrosis

**DOI:** 10.3389/fspor.2026.1786911

**Published:** 2026-03-27

**Authors:** Matthias Welsner, Jin-Sun Schermaul, Jose Guillermo Ortiz, Liron Lechtenberg, Christian Taube, Florian Stehling, Wolfgang Gruber

**Affiliations:** 1 Department of Pulmonary Medicine, University Hospital Essen - Ruhrlandklinik, Adult Cystic Fibrosis Center, University of Duisburg-Essen, Essen, Germany; 2 Pediatric Pulmonology and Sleep Medicine, Cystic Fibrosis Center, Childreńs Hospital, University of Duisburg-Essen, Essen, Germany

**Keywords:** adults, cystic fibrosis, mediation analysis, physical activity, physical activity enjoyment

## Abstract

**Background:**

This study aimed to investigate the mediating effect of physical activity enjoyment (PAE) on various physical activity (PA) levels in adult people with cystic fibrosis (pwCF) concerning disease-specific and non-disease-specific factors.

**Methods:**

A total of 168 adult pwCF (39.3% females; mean age 36.7 ± 11.9 years) completed questionnaires assessing PAE (Physical Activity Enjoyment Scale, PACES) and PA levels (7-day Physical Activity Recall, PAR). Participants’ demographics (age, sex) and clinical characteristics [ppFEV1; percent predicted forced expiratory volume in 1 s, and BMI; body mass index (kg/m2)]) were extracted from medical records. Mediation analysis was used to examine the direct and indirect effects of disease-specific and non-disease-specific factors on PA, considering PAE as a potential mediator.

**Results:**

Correlation analyses indicated a weak but statistically significant association between ppFEV1 and very vigorous physical activity (VVPA) (r = 0.177; *p* = 0.021) and PAE (r = 0.221; *p* = 0.004). PAE was linked to time spent in vigorous physical activity (VPA) (r = 0.202; *p* = 0.009), VVPA (r = 0.238; *p* = 0.002), and moderate-to-vigorous physical activity (MVPA) (r = 0.152; *p* = 0.049). Mediation analysis revealed that PAE fully mediated the association between lung function (ppFEV1) and vigorous PA (VPA), very vigorous PA (VVPA), and moderate-to-vigorous PA (MVPA) but not moderate PA (MPA). PpFEV1 and PAE together accounted for 26–49.3% of the variance in PA, suggesting the presence of other influential factors. No mediation effect was observed between PAE and age and BMI on any of the PA levels

**Conclusions:**

Our findings underscore the importance of both physiological and psychological factors in shaping PA among adults with CF. Beyond traditional clinical management, strategies that enhance PAE may be crucial for promoting sustained PA engagement. Future research should examine additional psychological and environmental factors influencing PAE and develop comprehensive approaches to support active lifestyles in this population.

## Background

Physical activity (PA) plays a crucial role in the non-pharmacological care of people with cystic fibrosis (pwCF). Lower PA levels are associated with decreased pulmonary function, impaired glycemic control, and reduced bone mineral density (1). Therefore, high levels of PA and fitness are associated with improved health-related quality of life (HrQoL), potentially slowing disease progression, and are therefore identified as prognostic factors (2, 3).

Despite its importance, the majority of pwCF do not engage in the recommended amounts of moderate-intensity PA of 150 min per week, or at least 75 min per week of vigorous-intensity physical activity, or an equivalent combination of both (3, 4). For additional health benefits, adults should increase their moderate-intensity PA to 300 min per week or equivalent (5). Studies on both pwCF and healthy populations have shown that extended periods of reduced moderate-to-vigorous physical activity (MVPA) result in an earlier onset of obesity and adult non-communicable diseases, such as cardiovascular and metabolic diseases (3, 6). Particularly in view of the increasing life expectancy of pwCF due to improved and new therapies, maintaining or increasing MVPA appears to be a preventive measure to avoid lifestyle diseases.

PwCF presents numerous challenges for individuals seeking to maintain an active lifestyle. Beyond the physical limitations imposed by the disease itself, such as impaired lung function, low muscle mass and strength, and physical symptoms (e.g., breathlessness, cough, and fatigue), pwCF often face a range of practical and psychological barriers to PA. In a systematic review by Denford et al. regarding barriers and facilitators to PA among pwCF, the feeling of “fun” and “joy” appears to be an important factor for sustained PA and has been identified as particularly significant in pwCF (7). Additional barriersinclude limited access to specialized equipment and professional guidance, social anxieties, lack of knowledge about safe exercise practices, and the unpredictable nature of symptom flare-ups (7–10). Moreover, medication side effects can further complicate efforts to engage in regular PA.

Most studies examining PA in pwCF are limited to general correlates, such as sex, anthropometric parameters, and age, as well as disease-specific correlates, including lung function and exercise tolerance (3). It is important to consider that PA is a complex, multidimensional behavior influenced by numerous internal (e.g., motivation, self-efficacy, health status, and enjoyment) and external factors (e.g., facility access, climate, and cultural norms) (11). The interaction between internal and external factors creates a dynamic system that affects PA patterns.

In general, enjoyment, as an intrinsic factor, is pivotal in fostering PA. When individuals find pleasure in PA, they are more likely to develop a long-term commitment to an active lifestyle (12). This positive emotional response creates a self-reinforcing cycle in which physical activity enjoyment (PAE) encourages continued participation, leading to improved physical fitness and overall well-being. Individuals who enjoy PA are more likely to seek out similar activities, explore diverse forms of exercise, and develop a positive attitude towards physical challenges. This intrinsic motivation helps overcome barriers to PA, as anticipated enjoyment becomes an incentive for participation. Furthermore, enjoyment of PA can foster social connections, enhance self-efficacy, and contribute to a positive body image, reinforcing the maintenance of an active lifestyle.

This study aimed to investigate the mediating effect of PAE on various PA levels concerning disease-specific and non-disease-specific factors within an adult CF population. We hypothesized that PAE significantly influences both the quantity and intensity of PA in adults with CF.

## Methods

### Subject selection and study design

The eligibility criteria for the study were as follows: participants must have a diagnosis of CF, be at least 18 years of age, and possess adequate German language proficiency. Additionally, candidates needed to be free from significant psychiatric or neurocognitive disorders and demonstrate sufficient cognitive capacity to comprehend and complete the questionnaires without substantial difficulty, as determined by the treating physician's clinical assessment.

All participants provided written informed consent before completing the questionnaires. The study followed the ethical principles of the Declaration of Helsinki and was approved by the local ethics committee of the University Hospital Essen (BO-24-11951). Participants’ demographics and clinical characteristics, including CF-specific data, lung function (ppFEV1; percent predicted forced expiratory volume in 1 s), and body mass index (BMI; kg/m^2^), were extracted from electronic medical records at the time nearest to the completion of the questionnaires.

The German version of the Physical Activity Enjoyment Scale (PACES) questionnaire was used to assess the extent to which individuals enjoy participating in PA (13). This instrument features 16 items, each evaluated on a 5-point Likert scale ranging from 1 (complete disagreement) to 5 (total agreement). The total score ranges from 16 to 80 points, with higher scores indicating greater enjoyment. The PACES questionnaire has demonstrated both reliability and validity in assessing PAE (14).

To evaluate regular PA patterns, we employed the 7-day Physical Activity Recall (PAR) questionnaire, a frequently used self-reporting tool. This instrument collects data on both PA and sedentary behavior from the previous week. The PAR questionnaire assesses the time (min/week) spent on various intensities of PA, including sleep, moderate physical activity (MPA), vigorous physical activity (VPA), and very-vigorous physical activity (VVPA) (15). The duration of moderate-to-vigorous PA (MVPA) was calculated as the sum of MPA and VPA durations. Its validity has been previously tested, even among pwCF, against objective measures, with generally positive results, leading to a consensus that it is a valid instrument for the measurement of PA (16–18).

To identify factors that influence or interact with PAE, participants were categorized into groups based on age (18-30, >30-45, and ≥45 years), ppFEV1 (<40, 40-69, and ≥70), BMI (<25, ≥25), and sex (male/female).

### Statistical analysis

Statistical analyses were performed using GraphPad v10.5. (GraphPad Software, Inc., La Jolla, California, USA). Data are presented as mean ± standard deviation (SD). The Shapiro–Wilk test was used to test for normal data distribution. Depending on the data distribution, either the Student's t-test or the Mann–Whitney U test was utilized to compare two groups (BMI, sex). For the comparison of three groups (ppFEV1, age), either an Analysis of Variance (ANOVA) or the Kruskal–Wallis test was conducted. *post-hoc* analysis was conducted using either the Bonferroni or Dunn tests, as appropriate.

**Figure 1:**
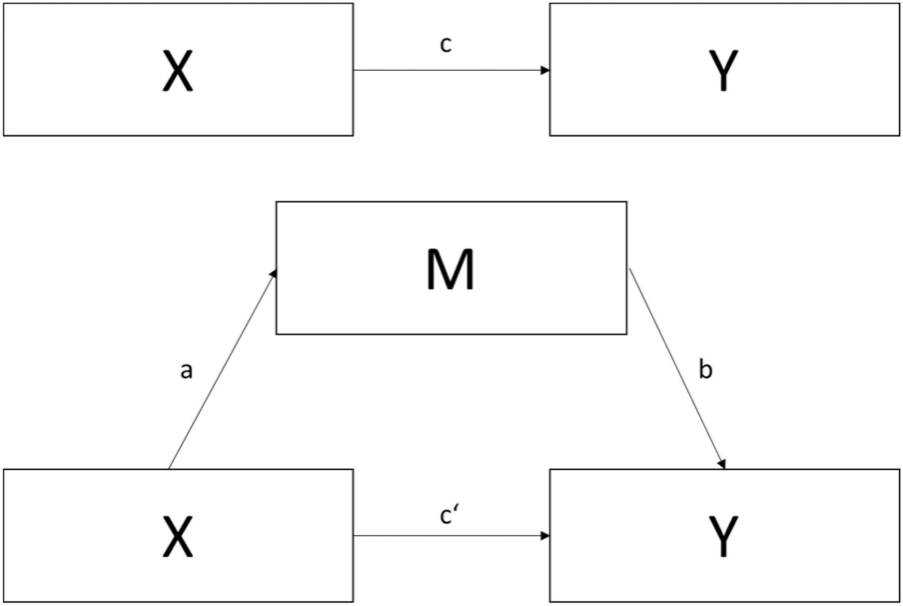
A conceptual model of a simple mediation model. X: dependent (predictor) variable. M: mediator. Y: independent (outcome) variable. a: effect of X on M. b: effect of M on Y. c′: direct effect of X on Y (controlling for M). ab: indirect effect of X on Y through M. c = ab + c′: total effect of X on Y.

Mediation analysis was performed using jamovi (version 2.6) (19). This approach allowed for the examination of both the direct and indirect effects of disease-specific and non-disease-specific factors on PA, considering PAE as a potential mediator (Figure 1).

For all analyses, a *p*-value ≤ 0.05 was considered statistically significant.

## Results

### Sample description

**Figure 2:**
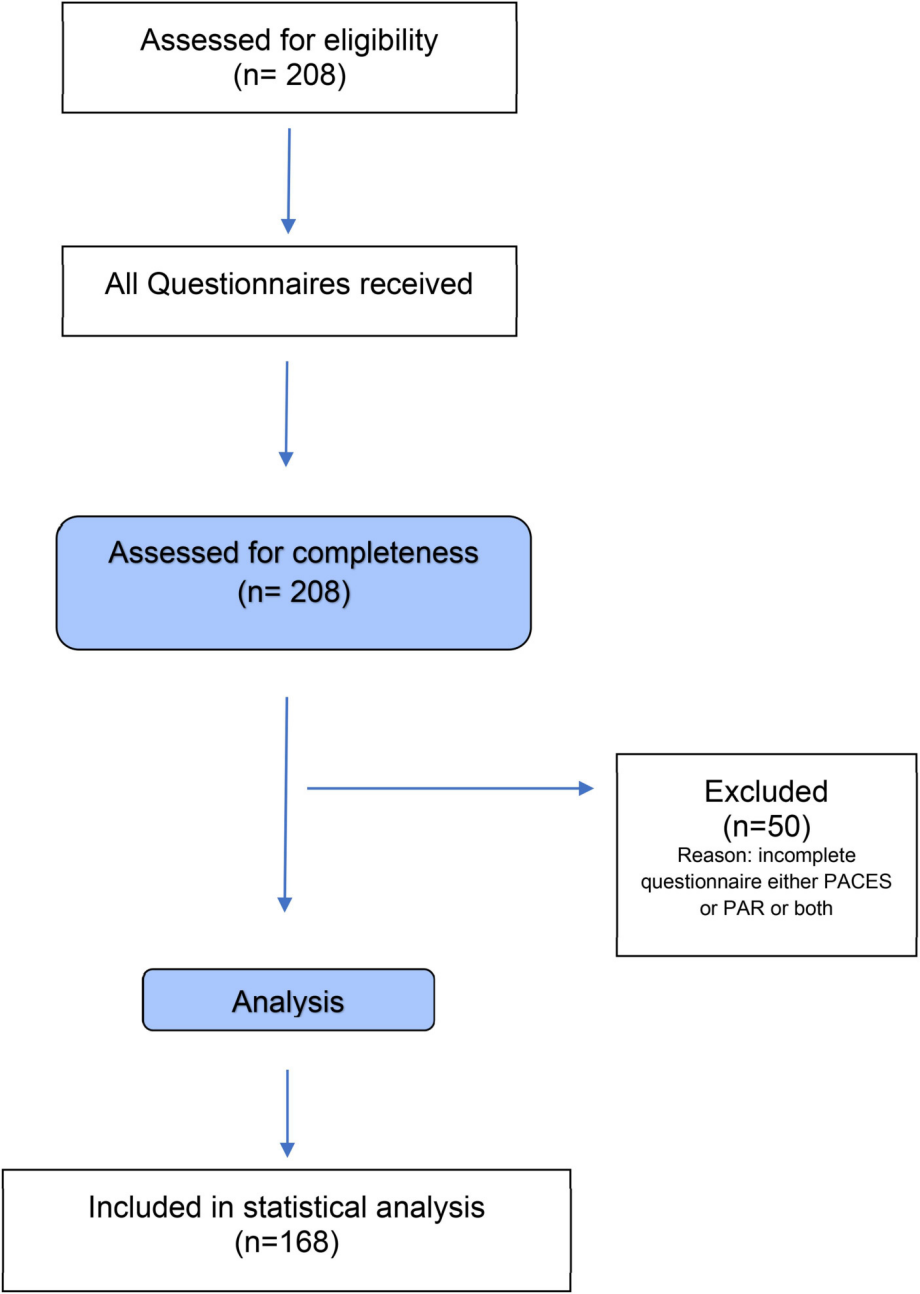
Participant flow diagram. PAR, 7-day physical activity recall; PACES, physical activity enjoyment scale.

A total of 208 pwCF participated in this study. After verifying the completeness of the questionnaires, 168 complete datasets (39.3% females) were deemed suitable for analysis (Figure 2). The mean age of the participants was 36.7 ± 11.9 years, with a mean ppFEV1 of 70.2 ± 22.4 and a BMI of 24.1 ± 4.5. Most pwCF were homozygous for the F508del mutation (46.4%). Of the 168 participants, 139 (83%) received CFTR modulator treatment with elexacaftor-tezacaftor-ivacaftor (ETI). The time interval between the clinical assessment (ppFEV1 and BMI) and the completion of the survey was 0.38 ± 1.85 days. A comprehensive overview of the participants’ demographics and clinical characteristics is presented in Table 1.

**Table 1:** Participitants’ demographics and clinical characteristics.

Characteristics	*N* = 168
Age (years)	36.7 ± 11.9 [34.9 - 38.5]
Female sex, n (%)	66 (39.3)
Genotype, n (%)	
F508del homozygous	78 (46.4)
F508del heterozygous	74 (44.1)
Other	16 (9.5)
CFTR modulator therapy, n (%)	
ETI	139 (82.7)
Mono-/dual	7 (4.2)
None	22 (13.1)
ppFEV1	70.2 ± 22.4 [66.8 - 73.6]
FEV1 [L]	2.7 ± 1.1 [2.6–2.9]
ppFVC	87.2 ± 18.6 [84.3–90.0]
FVC [L]	4.1 ± 1.2 [3.9–4.3]
Body weight [kg]	71.6 ± 15.3 [69.2–73.9]
BMI [kg/m^2^]	24.1 ± 4.5 [23.4 - 24.7]
Oxygen supplementation, n (%)	13 (7.7)
Pseudomonas aeruginosa, n (%)	85 (50.6)
Pancreatic insufficiency, n (%)	149 (88.7)
Cystic fibrosis-related diabetes, n (%)	16 (9.5)
PAE (PACES score)	65.4 ± 11.3 [63.7–67.2]
MPA (min/week)	656.3 ± 591.7 [566.2–746.5]
VPA (min/week)	216.6 ± 275.7 [174.6–258.6]
VVPA (min/week)	179.0 ± 248.4 [141.2–216.9]
MVPA (min/week)	827.9 ± 725.4 [762.4–983.4]

Values are expressed as mean ± standard deviation or number of patients (%). Brackets indicate the 95% confidence intervals (CI).

BMI, body mass index; ppFEV1, percent predicted forced expiratory volume in one second; ppFVC, percent predicted Forced Vital Capacity; CFTR, cystic fibrosis transmembrane conductance regulator, MPA, moderate physical activity; VPA, vigorous physical activity; VVPA, very vigorous physical activity; MVPA, moderate-to-vigorous physical activity; PAE, physical activity enjoyment; ETI, elexacaftor, tezacaftor, ivacaftor.

### Group differences physical activity enjoyment (PAE, PACES score)

**Figure 3:**
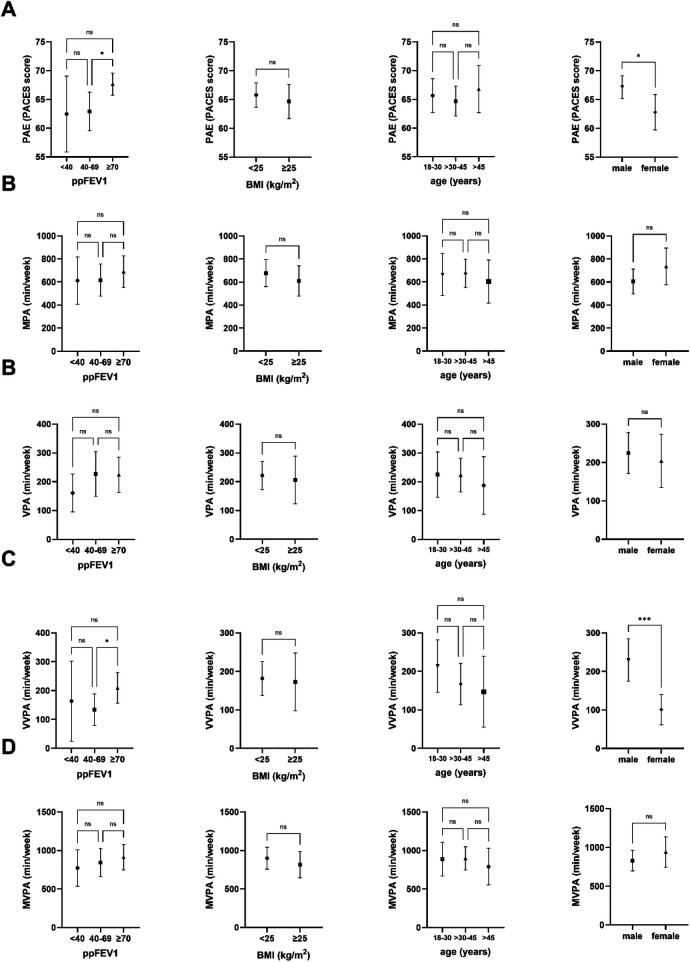
Group differences for PAE **(A)** and different PA levels **(B-D)**. Distribution of patient numbers **(n)** across the groups. Stratification includes: ppFEV1 < 40 (*n* = 22), 40–69 (*n* = 54), ≥70 (*n* = 92); BMI <25 (*n* = 116), ≥25 (*n* = 52); age 18–30 (*n* = 58), >30–45 (*n* = 76), >45 (*n* = 34); and sex (male, *n* = 102; female, *n* = 66). BMI, body mass index; ppFEV1, percent predicted forced expiratory volume in one second; MPA, moderate physical activity; VPA, vigorous physical activity; VVPA, very vigorous physical activity; MVPA, moderate-to-vigorous physical activity; PAE, physical activity enjoyment. * *p* < 0.05, ** *p* < 0.01, *** *p* < 0.001, NS, non-significant.

For a complete overview of the group differences for PAE, see Figure 3.

Female pwCF exhibited lower PAE levels than their male counterparts (*p* = 0.033). Those pwCF with preserved lung function (ppFEV1 ≥ 70) demonstrated a higher PACES score than those with a mildly reduced ppFEV1 of 40-69 (*p* = 0.041, but not those with ppFEV1 < 40 (*p* = 0.151). No significant group differences were observed in age (*p* = 0.569) and BMI (*p* = 0.349).

### Group differences PA levels

Figure 3 presents a comparative analysis of the groups concerning the different PA levels. Male pwCF demonstrated a significantly greater duration of VVPA than their female counterparts (*p* < 0.001). Additionally, no significant differences in VVPA were identified concerning ppFEV1, BMI, and age (all *p* > 0.05). Subgroup analyses revealed that pwCF with ppFEV1 ≥ 70 engaged in more VVPA than those with ppFEV1 ranging from 40 to 69 (*p* = 0.039) but not those with ppFEV1 < 40 (*p* = 0.074). No significant differences were observed among the groups for MPA, VPA, or MVPA (all *p* > 0.05).

### Correlation analysis

As shown in Table 2, the correlation analyses indicated a weak but statistically significant association between ppFEV1 and both VVPA (r = 0.177; *p* = 0.021) and PAE (r = 0.221; *p* = 0.004). In contrast, no significant correlation was observed between ppFEV and MPA (r = 0.056; *p* = 0.469), VPA (r = 0.086; *p* = 0.267), or MVPA (r = 0.079; *p* = 0.311). PAE was linked to the time spent in VPA (r = 0.202; *p* = 0.009), VVPA (r = 0.238; *p* = 0.002), and MVPA (r = 0.152; *p* = 0.049), but not with MPA (r = 0.092; *p* = 0.236). Additionally, there was no correlation found between BMI or age and PA levels (all *p* > 0.05).

**Table 2:** Correlation matrix.

Variable	Correlation coefficient p-value	Age (years)	BMI (kg/m^2^)	ppFEV1	MPA (min/week)	VPA (min/week)	VVPA (min/week)	MVPA (min/week)
**MPA** **(min/week)**	Pearson's r	-0.008	0.001	0.056	—			
	p-value	0.915	0.988	0.469	—			
**VPA** **(min/week)**	Pearson's r	-0.085	-0.075	0.086	0.307***	—		
	p-value	0.275	0.336	0.267	<.001	—		
**VVPA** **(min/week)**	Pearson's r	-0.056	-0.021	0.177*	0.040	0.261***	—	
	p-value	0.472	0.791	0.021	0.608	<.001	—	
**MVPA** **(min/week)**	Pearson's r	-0.039	-0.027	0.079	0.932***	0.630***	0.132	—
	p-value	0.616	0.724	0.311	<.001	<.001	0.089	—
**PAE** **(PACES score)**	Pearson's r	-0.003	-0.012	0.221**	0.092	0.202**	0.238**	0.152*
	p-value	0.964	0.874	0.004	0.236	0.009	0.002	0.049

BMI, body mass index; ppFEV1, percent predicted forced expiratory volume in one second; MPA, moderate physical activity; VPA, vigorous physical activity; VVPA, very vigorous physical activity; MVPA, moderate-to-vigorous physical activity, PAE, physical activity enjoyment.

*p < 0.05, **p < 0.01, ***p < 0.001, NS, non-significant.

### Mediation analysis

Mediation analysis supported the hypothesis that PAE mediates the relationship between ppFEV1 and PA (Table 3). PpFEV1 demonstrated a positive association with PAE at different PA levels (MPA: a = 0.111, *p* = 0.010; VPA: a = 0.111, *p* = 0.009, VVPA: a = 0.111, *p* = 0.008 and MVPA: a = 0.111, *p* = 0.009), and PAE was positively correlated with time spent in different PA levels: VPA (b = 4.639, *p* = 0.002), VVPA (b = 4.594, *p* < 0.001, and MVPA (b = 9.063, *p* = 0.025), but not to MPA (b = 4.369, *p* = 0.174). A bootstrap interval with 5000 repetitions for the indirect effect (ab) was entirely above zero for VPA [ab = 0.523, 95%CI (0.100, 1.13)], VVPA [ab = 0.512, 95%CI (0.112, 1.04)], and MVPA [ab = 1.01, 95%CI (0.0384, 2.49)], indicating that PAE fully mediates the association between ppFEV1 and VPA, VVPA, and MVPA, but not with MPA [ab = 0.487; 95%CI (−0.152, 1.56)]. No mediation effect was observed between PAE and age and BMI on any of the PA levels (Supplement 1 and 2).

**Table 3:** Results of a simple mediation analysis with a.) MPA, b.) VPA, c.) VVPA and d.) MVPA as dependent variable (X), ppFEV1 as independent variable (Y) and PAE as mediator variable (M).

a.) Mediation analysis for ppFEV1 (X), MPA (Y) and PAE (M)									
Mediation Estimates									
95% Confidence Interval									
Effect	Label	Estimate	SE	Lower	Upper	Z	p	% Mediation	
Indirect	a × b	0.487	0.445	−0.152	1.56	1.095	0.274	32.7	
Direct	c	1.001	1.926	−2.773	4.80	0.519	0.603	67.3	
Total	c + a × b	1.488	1.849	−2.015	5.19	0.805	0.421	100.0	
Path Estimates									
95% Confidence Interval									
			Label	Estimate	SE	Lower	Upper	Z	p
ppFEV1	→	PAE	a	0.111	0.0433	0.0254	0.195	2.575	0.010
PAE	→	MPA (min/week)	b	4.369	3.2111	−1.7040	10.958	1.361	0.174
ppFEV1	→	MPA (min/week)	c	1.001	1.9265	−2.7728	4.797	0.519	0.603
b.) Mediation analysis for ppFEV1 (X), VPA (Y) and PAE (M)									
Mediation Estimates									
				95% Confidence Interval					
Effect	Label	Estimate	SE	Lower	Upper	Z	p	% Mediation	
Indirect	a × b	0.523	0.268	0.100	1.13	1.955	0.051	49.3	
Direct	c	0.537	0.947	−1.175	2.52	0.567	0.571	50.7	
Total	c + a × b	1.060	0.899	−0.615	2.93	1.179	0.238	100.0	
Path Estimates									
						95% Confidence Interval			
			Label	Estimate	SE	Lower	Upper	Z	p
ppFEV1	→	PAE	a	0.111	0.0426	0.0283	0.196	2.617	0.009
PAE	→	VPA (min/week)	b	4.693	1.5030	1.7283	7.660	3.123	0.002
ppFEV1	→	VPA (min/week)	c	0.537	0.9473	−1.1748	2.522	0.567	0.571
c.) Mediation analysis for ppFEV1 (X), VVPA (Y) and PAE (M)									
Mediation Estimates									
				95% Confidence Interval					
Effect	Label	Estimate	SE	Lower	Upper	Z	p	% Mediation	
Indirect	a × b	0.512	0.237	0.11225	1.04	2.16	0.030	26.0	
Direct	c	1.457	1.004	−0.49982	3.47	1.45	0.146	74.0	
Total	c + a × b	1.970	0.992	−0.00243	3.98	1.99	0.047	100.0	

Path Estimates									
						95% Confidence Interval			
			Label	Estimate	SE	Lower	Upper	Z	p
ppFEV1	→	PAE	a	0.111	0.0421	0.0301	0.193	2.65	0.008
PAE	→	VVPA (min/week)	b	4.594	1.1617	2.3031	6.906	3.96	<.001
ppFEV1	→	VVPA (min/week)	c	1.457	1.0037	−0.4998	3.469	1.45	0.146
d.) Mediation analysis for ppFEV1 (X), MVPA (Y) and PAE (M)									
Mediation Estimates									
				95% Confidence Interval					
Effect	Label	Estimate	SE	Lower	Upper	Z	p	% Mediation	
Indirect	a × b	1.01	0.640	0.0384	2.49	1.578	0.115	39.7	
Direct	c	1.54	2.368	−2.9694	6.28	0.649	0.516	60.3	
Total	c + a × b	2.55	2.252	−1.6915	7.06	1.131	0.258	100.0	
Path Estimates									
						95% Confidence Interval			
			Label	Estimate	SE	Lower	Upper	Z	p
ppFEV1	→	PAE	a	0.111	0.0427	0.0271	0.197	2.610	0.009
PAE	→	MVPA (min/week)	b	9.063	4.0395	1.0253	16.957	2.244	0.025
ppFEV1	→	MVPA (min/week)	c	1.538	2.3683	−2.9694	6.278	0.649	0.516

ppFEV1, percent predicted forced expiratory volume in one second; MPA, moderate physical activity; VPA, vigorous physical activity; VVPA, very vigorous physical activity; MVPA, moderate-to-vigorous physical activity, PAE, physical activity enjoyment.

## Discussion

Our data indicate that among adult pwCF, the extent and intensity of PA are significantly influenced by pulmonary function (ppFEV1) and PAE. Notably, mediation analysis revealed that PAE fully mediated the effect of ppFEV1 on PA, especially at higher PA levels, underscoring the central role of affective factors in PA engagement. Conversely, age and nutritional status (BMI) played only minor roles and did not significantly affect PA levels in our cohort.

A detailed comparison of our findings with the literature reveals both convergences and notable divergences regarding the determinants of PA in adult pwCF. Consistent with established research, our data corroborate the positive association between pulmonary function (ppFEV1) and engagement in higher-intensity physical activities (20, 21). This alignment suggests that preserved lung function remains a physiological prerequisite for vigorous exertion, likely due *to the reduced ventilatory limitation and enhanced exercise capacity characteristic of higher ppFEV1 values* (2)*. However, our study extends these observations by elucidating the psychological mechanisms underpinning this relationship. While previous studies have largely treated ppFEV1 as a direct correlate of PA behavior* (3)*, our mediation analysis demonstrates that this association is not merely physiological but is substantially channeled through PAE. This finding suggests that the pathway from better lung function to higher activity levels is, to a significant extent, affectively mediated; individuals with superior lung function may derive greater* enjoyment from strenuous activities, thereby reinforcing their participation. This affective mediation aligns with self-determination theory (SDT), which posits that intrinsic motivation - driven by enjoyment and perceived competence - is critical for sustained PA, especially in chronic illness populations (22, 23).

In contrast to the strong link between ppFEV1 and vigorous PA, the absence of a significant association between ppFEV1 and MPA in our cohort warrants discussion. This divergence from some prior reports, which have indicated correlations across all intensity levels, may reflect the unique behavioral patterns of the modern CF population, particularly in the context of effective CFTR-modulator therapies (24, 25). It is plausible that while reduced lung function imposes a ceiling on vigorous exertion, it does not necessarily preclude engagement in moderate-intensity activities. Consequently, the variance in MPA may be driven more by environmental or motivational factors rather than physiological capacity, a hypothesis supported by the lack of mediation by PAE in this specific intensity domain.

Furthermore, the identification of PAE as a full mediator for vigorous activities aligns with, yet theoretically advances, the findings of Denford et al. (7), who identified ‘fun’ and ‘joy’ as qualitative facilitators. Our study quantifies this concept, demonstrating that PAE is not merely a peripheral benefit but a central, independent determinant of PA behavior. This is particularly relevant when contrasted with studies in healthy populations, where the relationship between physiological capacity and PA is often more direct (26). In the context of chronic disease, where physical limitations are omnipresent, the affective response to exercise appears to gain prominence as a regulatory factor for engagement (27). The lack of influence from age and BMI observed in our study further distinguishes our cohort from general population data, where these demographic factors typically show stronger inverse correlations with PA levels (28). This suggests that in pwCF, the disease-specific physiological and psychological variables may overshadow the general age-related decline in activity often seen in healthy adults.

However, PAE and ppFEV1 together accounted for only 26–49.3% of the variance in PA intensity and extent, suggesting that other factors are also influential. Both our results and previous studies indicate that other psychological variables, such as self-efficacy, motivation, and symptoms of anxiety or depression, can substantially affect PA engagement in pwCF (10, 29, 30).

The introduction of highly effective CFTR modulator therapies (HEMT) has significantly altered the trajectory of the disease, yielding a multitude of benefits for individuals afflicted with this genetic disorder. Notably, these therapies have led to clinical stabilization, as evidenced by improvements in pulmonary function, nutritional status, and reduction in exacerbation rates (31). Furthermore, the enhanced quality of life (HrQoL) associated with these treatments has become a hallmark of CF care (32). The high proportion of pwCF receiving HEMT in our cohort (83%) likely played a significant role in the observed PA levels. While medication use was not included as a separate mediator in our analysis due to its strong correlation with lung function, we acknowledge that CFTR modulators act as a key facilitator by improving clinical stabilization and physical health. This pharmacological benefit may create the physiological prerequisite that allows patients to engage in and enjoy physical activity, thereby interacting with the psychological factors examined in this study.

However, as the disease's management has become increasingly effective, a shift in focus has occurred, with comorbidities such as overweight and obesity gaining prominence. The favorable conditions created by CF modulator therapies, which enable individuals to maintain or increase their PA, have inadvertently contributed to an increased risk of obesity (33, 34). Consequently, it is essential to address this emerging concern and develop targeted interventions to mitigate the risk of lifestyle-related diseases (35). As the physical limitations associated with CF recede, other factors influencing PA emerge. Self-efficacy, motivation, enjoyment, and environmental factors are increasingly recognized as crucial determinants of PA levels in individuals with CF (36). Therefore, a holistic approach to PA promotion becomes more important in future.

Our data underscore a distinct need for such enjoyment-focused strategies. With a mean PACES score of 65.4 ± 11.3, enjoyment levels in our cohort were only moderate, suggesting that current standard care does not fully exploit the potential of intrinsic motivation. Furthermore, given that our mediation analysis established PAE as a full mediator for vigorous PA, targeting these psychological barriers is not optional but essential. Interventions that leverage social engagement, supervision, and virtual elements are therefore directly supported by our finding that enhancing the affective response to exercise is a key mechanism to increase activity levels, independent of clinical status. To adapt strategies for enhancing PAE in pwCF, several key approaches warrant consideration. Leveraging social engagement and group dynamics is essential, as social interaction significantly enhances enjoyment by fostering accountability, camaraderie, and a sense of belonging (37).

Guidance and organized assistance can significantly contribute to improving PAE (38). Supervised exercise programs have been particularly successful in enhancing outcomes for pwCF, as they offer a secure and supportive setting in which pwCF can participate in regular PA under the supervision of trained healthcare professionals (39, 40). These programs provide numerous benefits, including better lung function, increased strength and endurance, fewer respiratory symptoms, improved mental health, such as reduced anxiety and depression, and better adherence to exercise, which is essential for sustaining these advantages (41). One major benefit of supervision is the promotion of positive social interaction. When individuals engage in PA with the support and encouragement of a supervisor, they are more likely to feel a sense of community and belonging. Another significant advantage of supervision is personalized instruction. When individuals receive customized guidance and feedback from a supervisor, they tend to feel more confident and capable in their PA. This can result in increased PAE and a lower risk of injury, as individuals can learn and refine their techniques in a safe, supportive environment. In addition to the benefits of positive social interaction and personalized instruction, supervision can help reduce anxiety related to trying new activities or pushing physical limits.

A key strength of this study lies in the application of mediation analysis, which offers distinct advantages over simple correlation approaches. While standard correlations can confirm that lung function is associated with PA, they do not elucidate the mechanisms driving this relationship. By employing mediation analysis, we were able to decompose the effect of ppFEV1 on PA into direct and indirect components. This allowed us to demonstrate that the influence of lung function on vigorous activity is largely transmitted through the psychological pathway of enjoyment, providing deeper insight into the behavioral drivers in adults with CF than associative measures alone could offer. Virtual group workouts, such as online cycling classes or fitness challenges, have gained popularity, offering flexibility while maintaining social connections (42). Digital tools and gamification elements such as rewards and progress tracking can make PA more engaging (43, 44). Virtual reality (VR) workouts, such as immersive cycling or dance games, have also shown promise in enhancing enjoyment by making exercise feel like a game (45). Monotony is a common barrier to enjoyment, and introducing variety, such as alternating between cardio, strength training, and flexibility exercises, can sustain interest (46). Emphasizing the immediate non-physical benefits of PA, such as stress relief, mental clarity, and emotional well-being, can enhance intrinsic motivation (47).

This study has several limitations. First, reliance on self-reported measures introduces potential recall and social desirability biases; more objective measures, such as accelerometry, would strengthen future work. Second, recruitment from a single center may limit generalizability; thus, multicenter or international samples are recommended for future studies. Third, although we included several relevant covariates, unmeasured factors such as specific psychological traits, the extent of social support, and environmental considerations may have impacted our findings. Because PAE and ppFEV1 explained only a modest proportion of the variance in PA, it is evident that additional, as yet unexamined, factors contribute to PA levels in this group.

## Conclusions

In conclusion, both physiological (ppFEV1) and psychological (PAE) factors play significant roles in shaping PA among adult pwCF. Beyond traditional clinical management, strategies that enhance PAE may be crucial for promoting sustained engagement. The limited explanatory power of ppFEV1 and PAE underscores the necessity of investigating additional psychological and environmental factors that influence PAE in pwCF. Future research should further examine these factors and develop comprehensive approaches to support active lifestyles in this unique population.

## Data Availability

The raw data supporting the conclusions of this article will be made available by the authors, without undue reservation.
