# Supplementary material for: Mediating effects of physical activity enjoyment on physical activity levels in adults with cystic fibrosis
Source: Front Sports Act Living. 2026 Mar 27;8:1786911. doi: 10.3389/fspor.2026.1786911 (PMC13066178; doi:10.3389/fspor.2026.1786911)
Supplement: Supplementary file 1 [file Table1.docx]

**Supplement 1.**

Results of a simple mediation analysis with a.) MPA, b.) VPA, c.) VVPA and d.) MVPA as dependent variable (X), BMI as independent variable (Y) and PAE as mediator variable (M)

a.) Mediation analysis for BMI (X), MPA (Y) and PAE (M)

| Mediation Estimates | | | | | | | | |
| --- | --- | --- | --- | --- | --- | --- | --- | --- |
|  | | | | **95% Confidence Interval** | |  | | |
| **Effect** | **Label** | **Estimate** | **SE** | **Lower** | **Upper** | **Z** | **p** | **% Mediation** |
| Indirect | a × b | -0.149 | 1.14 | -2.84 | 2.05 | -0.1306 | 0.896 | 32.9 |
| Direct | c | 0.304 | 8.70 | -14.62 | 19.78 | 0.0349 | 0.972 | 67.1 |
| Total | c + a × b | 0.155 | 8.59 | -14.37 | 19.43 | 0.0180 | 0.986 | 100.0 |

| Path Estimates | | | | | | | | | |
| --- | --- | --- | --- | --- | --- | --- | --- | --- | --- |
|  | | | | | | **95% Confidence Interval** | |  | |
|  |  |  | **Label** | **Estimate** | **SE** | **Lower** | **Upper** | **Z** | **p** |
| BMI | → | PAE | a | -0.0309 | 0.193 | -0.424 | 0.342 | -0.1607 | 0.872 |
| PAE | → | MPA (min/week) | b | 4.8075 | 3.131 | -1.334 | 11.023 | 1.5357 | 0.125 |
| BMI | → | MPA (min/week) | c | 0.3038 | 8.701 | -14.619 | 19.783 | 0.0349 | 0.972 |

b.) Mediation analysis for BMI (X), VPA (Y) and PAE (M)

| Mediation Estimates | | | | | | | | |
| --- | --- | --- | --- | --- | --- | --- | --- | --- |
|  | | | | **95% Confidence Interval** | |  | | |
| **Effect** | **Label** | **Estimate** | **SE** | **Lower** | **Upper** | **Z** | **p** | **% Mediation** |
| Indirect | a × b | -0.152 | 0.977 | -2.19 | 1.81 | -0.155 | 0.876 | 3.33 |
| Direct | c | -4.412 | 4.007 | -11.80 | 4.24 | -1.101 | 0.271 | 96.67 |
| Total | c + a × b | -4.564 | 4.141 | -12.24 | 4.50 | -1.102 | 0.270 | 100.00 |

| Path Estimates | | | | | | | | | |
| --- | --- | --- | --- | --- | --- | --- | --- | --- | --- |
|  | | | | | | **95% Confidence Interval** | |  | |
|  |  |  | **Label** | **Estimate** | **SE** | **Lower** | **Upper** | **Z** | **p** |
| BMI | → | PAE | a | -0.0309 | 0.191 | -0.418 | 0.331 | -0.162 | 0.871 |
| PAE | → | VPA (min/week) | b | 4.9061 | 1.381 | 2.207 | 7.617 | 3.554 | <.001 |
| BMI | → | VPA (min/week) | c | -4.4118 | 4.007 | -11.802 | 4.245 | -1.101 | 0.271 |

c.) Mediation analysis for BMI (X), VVPA (Y) and PAE (M)

| Mediation Estimates | | | | | | | | |
| --- | --- | --- | --- | --- | --- | --- | --- | --- |
|  | | | | **95% Confidence Interval** | |  | | |
| **Effect** | **Label** | **Estimate** | **SE** | **Lower** | **Upper** | **Z** | **p** | **% Mediation** |
| Indirect | a × b | -0.162 | 1.04 | -2.36 | 1.85 | -0.156 | 0.876 | 14.3 |
| Direct | c | -0.971 | 3.64 | -6.83 | 7.49 | -0.266 | 0.790 | 85.7 |
| Total | c + a × b | -1.133 | 3.77 | -7.35 | 7.58 | -0.301 | 0.764 | 100.0 |

| Path Estimates | | | | | | | | | |
| --- | --- | --- | --- | --- | --- | --- | --- | --- | --- |
|  | | | | | | **95% Confidence Interval** | |  | |
|  |  |  | **Label** | **Estimate** | **SE** | **Lower** | **Upper** | **Z** | **p** |
| BMI | → | PAE | a | -0.0309 | 0.194 | -0.437 | 0.336 | -0.160 | 0.873 |
| PAE | → | VVPA (min/week) | b | 5.2255 | 1.136 | 2.977 | 7.432 | 4.601 | <.001 |
| BMI | → | VVPA (min/week) | c | -0.9709 | 3.644 | -6.829 | 7.493 | -0.266 | 0.790 |

d.) Mediation analysis for BMI (X), MVPA (Y) and PAE (M)

| Mediation Estimates | | | | | | | | |
| --- | --- | --- | --- | --- | --- | --- | --- | --- |
|  | | | | **95% Confidence Interval** | |  | | |
| **Effect** | **Label** | **Estimate** | **SE** | **Lower** | **Upper** | **Z** | **p** | **% Mediation** |
| Indirect | a × b | -0.301 | 2.07 | -4.75 | 3.91 | -0.145 | 0.884 | 6.82 |
| Direct | c | -4.108 | 10.65 | -22.69 | 19.27 | -0.386 | 0.700 | 93.18 |
| Total | c + a × b | -4.409 | 10.48 | -22.64 | 18.70 | -0.421 | 0.674 | 100.00 |

| Path Estimates | | | | | | | | | |
| --- | --- | --- | --- | --- | --- | --- | --- | --- | --- |
|  | | | | | | **95% Confidence Interval** | |  | |
|  |  |  | **Label** | **Estimate** | **SE** | **Lower** | **Upper** | **Z** | **p** |
| BMI | → | PAE | a | -0.0309 | 0.194 | -0.419 | 0.343 | -0.160 | 0.873 |
| PAE | → | MVPA (min/week) | b | 9.7135 | 3.786 | 2.232 | 17.187 | 2.565 | 0.010 |
| BMI | → | MVPA (min/week) | c | -4.1080 | 10.649 | -22.686 | 19.267 | -0.386 | 0.700 |

Body Mass Index (BMI); MPA, moderate physical activity; VPA, vigorous physical activity; VVPA, very vigorous physical activity; MVPA,

moderate-to-vigorous physical activity, PAE, physical activity enjoyment.
